# Effectiveness of applying clinical simulation scenarios and integrating information technology in medical-surgical nursing and critical nursing courses

**DOI:** 10.1186/s12912-021-00744-7

**Published:** 2021-11-15

**Authors:** Li-Ping Tseng, Tung-Hsu Hou, Li-Ping Huang, Yang-Kun Ou

**Affiliations:** 1 Department of Management Center, Sisters of our Lady of China Catholic Medical Foundation, St. Martin De Porres Hospital, Chiayi City, 60069 Taiwan; 2 Department of Industrial Engineering and Management, National Yunlin University of Science and Technology, Yunlin, 640301 Taiwan; 3 Department of Nursing, Chung-Jen Junior College of Nursing, Health Sciences and Management, Chiayi, 60077 Taiwan; 4 Department of Creative Product Design, Southern Taiwan University of Science and Technology, No. 1, Nan-Tai Street, Yungkang Dist, Tainan City, 71005 Taiwan

**Keywords:** Clinical simulation scenario, Information technology integrated instruction (ITII), Objective structured clinical examination (OSCE), Situational awareness (SA), Innovative nursing teaching

## Abstract

**Background:**

To determine the impact of combining clinical simulation scenario training and Information Technology Integrated Instruction (ITII) on the teaching of nursing skills.

**Methods:**

120 4th-year students in a nursing program who were enrolled in medical and surgical nursing courses. 61 received innovative instruction (experimental group) and 59 received conventional instruction (control group). The ADDIE model, systematic method of course development that includes analysis, design, development, implementation, and evaluation,was used to build simulation teaching and clinical scenarios and to create and modify objective structure clinical examination (OSCE) scenario checklists for acute myocardial infarction (AMI) care, basic life support and operation of automated external defibrillator (BLS), and subdural hemorrhage (SDH) care. The modified OSCE checklists were assessed for reliability, consistency, and validity. The innovative training included flipped classrooms, clinical simulation scenarios, ITII and blended learning formats.

**Results:**

The reliability and validity of the OSCE checklists developed in this study were acceptable and comparable or higher than checklists in past studies and could be utilized as an OSCE performance tool. Students in innovative instruction obtained significantly better OSCE performance, lab scores and improvements from the previous year’s grades. Significant differences were found in situational awareness (SA). No strong correlations were found between OSCE scores and clinical internship scores, and no significant differences were found between the groups in overall clinical internship performance.

**Conclusions:**

Innovative instruction showed better performance than conventional methods in summative evaluation of knowledge components, OSCE formative evaluation and clinical nursing internship scores, as well as improved situational awareness in nursing students.

## Introduction

Nurses play a crucial role in the healthcare industry, offering comprehensive patient care and establishing close relationships with patients. High-quality clinical care is dependent on thorough training and assessment methods. Proper nursing education is essential. Objective structure clinical examinations (OSCE) were designed by Harden et al. in 1975 to evaluate the clinical competency and skills of graduating medical students and have since been adapted for nursing students as well. In OSCEs, students spend 5 min each at a series of test stations, where they are observed as they interact with clinical environments with either real patients or actors. OSCEs extensively test clinical skills, stimulate learning, and have been found to be less biased and have better reliability, validity, and objectivity than other evaluation methods [1–5]. Studies have shown that combining simulation-based education and OSCEs can reinforce the objectivity of clinical competency evaluations [6, 7]. Regardless, OSCEs have been verified as an effective tool and presented as an outcome-based test for evaluating students and their clinical performances after graduation [8]. In nursing education, OSCEs have become increasingly integrated with multiple disciplines and are used both as formative and summative evaluations. Therefore, this study utilized OSCE as a tool to evaluate student performance.

However, although OSCEs provide a way to evaluate and improve the transfer of classroom and lab learning into simulated clinical scenarios, some argue that it is inappropriate to assume that such simulation performance translates into real-world competence [7, 8]. This might be due to the uncertain and unpredictable nature of the care process itself. Therefore, integrating skills such as perception, comprehension and projection into OSCEs can further fortify the quality of care. Situational awareness (SA) refers to how people pay attention to surrounding events, the information they notice, and their use of that information to form plans or decisions [9, 10]. The three SA stages of cognitive performance are perception (SA1), comprehension (SA2), and projection (SA3). In SA1, people use sensory input to understand events occurring in the surrounding environment. In SA2, perceived information is processed, identifying relationships between clues and task goals, and fully understanding the situation. In SA3, predictions of future results are formed based on SA1 and SA2. The formulation of actions and strategies required in the future, which is based on the three stages, is the highest form of SA [9, 11–13]. These three stages are dynamic, and the appropriateness of a person’s current SA and the quality of their decision-making and performance depend on the task at hand and the individual and system factors involved, such as knowledge, perception, task goals, level of understanding, interpretation of messages, attention, and memory [14]. Nursing care is also a dynamic process, therefore nurses should be trained towards highest levels of SA to ensure the highest-quality patient outcomes and safety.

Student evaluations to assess evidence of learning are an essential part of systemic approaches to teaching [15]. Summative and formative evaluations reflect the realities, operational difficulties, and challenges of the teaching process to support learning in the future [16]. With the rapid development of the Internet, information skills have become key to students’ competitiveness. Online education – whether in the form of massive open online courses (MOOCs), small private online courses (SPOCs), interactive educational software, simulation devices, teaching platforms, sharing platforms, or otherwise – has subverted conventional teaching methods. As a result, continuous change and innovations such as flipped classrooms and blended teaching continue to challenge educational institutions at every level, as well instructors and students [17–20]. Wang and Zhu [21] compared MOOC-based flipped learning against conventional instruction in an inorganic chemistry course and found students in the former performing better than those in the latter; furthermore, students in the former found the MOOC-based curriculum helpful with the knowledge component of the course, but not in the practicum and feedback. Li, Zhang and Hu [22] explored flipped learning on Moodle on-line platform and found increased student autonomy, motivation and content knowledge. The ways in which technology can be effectively incorporated into nursing education merits research and discussion.

**Fig. 1:**
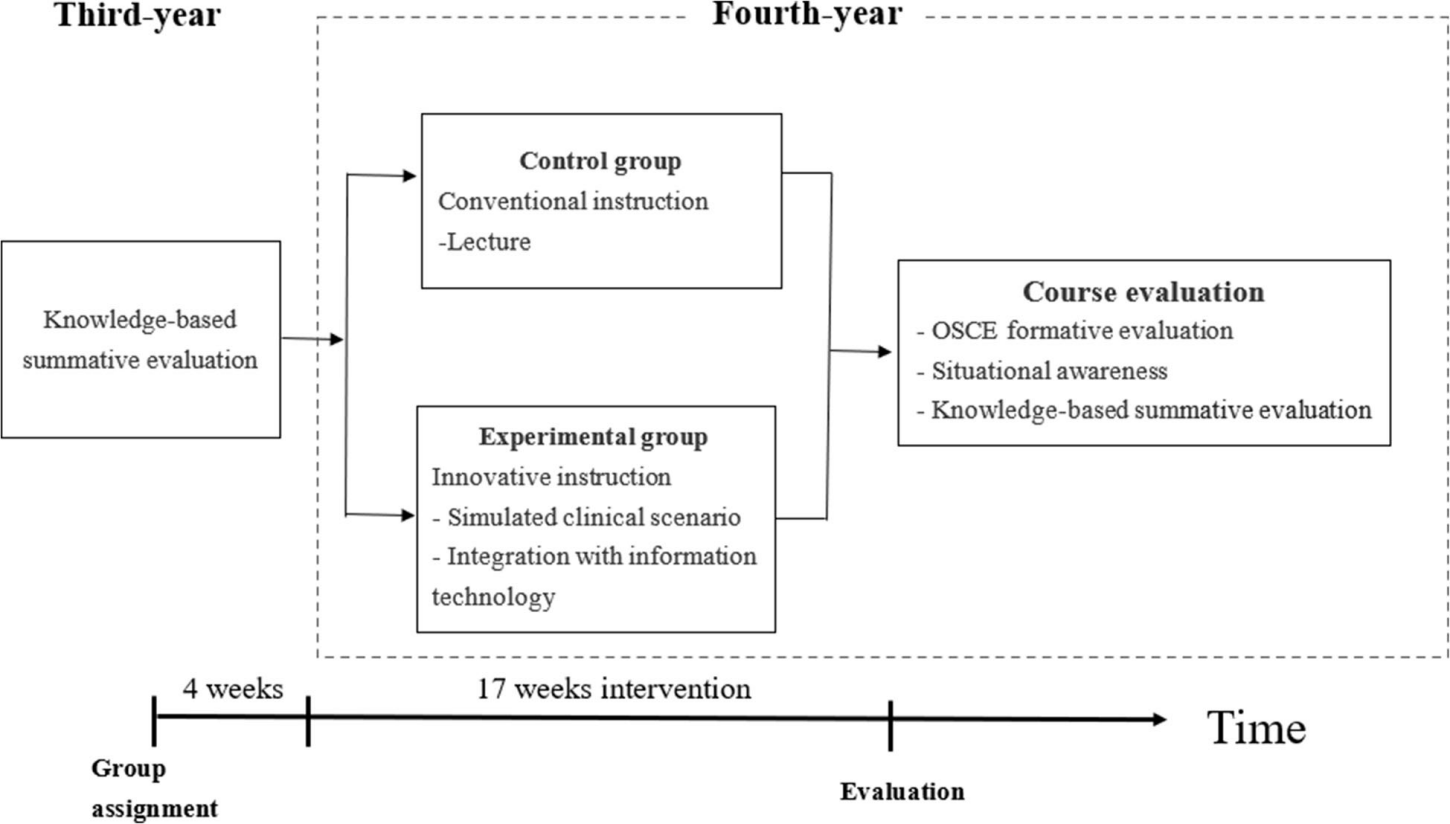
Conceptual Construct Diagram

Rather than one-way lecture in conventional nursing education, innovative instruction integrates technological products into teaching to promote interaction between instructors and students in order to boost student motivation and performance. The purpose of this study is to integrate applications and products such as SPOCs, Zuvio, advanced simulator, smart Little Anne QCPR manikin, AED, Moodle, LINE into nursing education strategies for internal medicine, surgical nursing, and critical care nursing courses for fourth-year students in 5-year Taiwanese junior college programs and to attempt to understand whether innovative instruction (in the form of information technology integrated instruction, ITII) made a difference on the effectiveness of OSCE, or has any effect on SA. The conceptual construct for this study is shown in Fig. 1. In summary, the purpose of this study is as follows:

1. To build clinical scenarios for medical and surgical nursing courses to be combined with OSCE, and to test the validity and reliability of the resulting OSCE checklist.
2. To investigate the correlations between the medical and surgical clinical internship scores and OSCE scores in innovative and conventional instruction
3. To investigate the differences in OSCE by innovative and conventional instruction.
4. To explore the differences in situational awareness between innovative and conventional instruction.
5. To explore how innovative and conventional instruction affect knowledge-based summative evaluations.

## Material and methods

### Participants

This study was approved by the Institutional Review Board of St. Martin De Porres Hospital (IRB No.18B-12). Using purposive sampling, this study recruited fourth-year students in a 5-year program at a medical and nursing management junior college in central Taiwan. Prior to the first semester of the 2019 academic year, fourth-year students who started in the program in 2016 were identified. Student recruits were informed in detail of the purpose and procedures of the study, as well as any potential risks and interests, and were given ample time to consider before signing consent. A total of 120 students were enrolled in the study, aged between 19 and 20 years, five were males and the remaining were females. Consent from legal guardians were obtained for participants under 20 years. All participants were given the right to terminate participation in the study at any time without any conditions.

### Study design

In order to compare student performance between conventional and innovative instruction, purposive sampling was used to divide the participants into experimental and control groups. Course contents of the two groups were similar, only the method of instruction differed – innovative instruction for the experimental group, and conventional for the control group. Within-group factors were simulated scenarios and levels of situational awareness. The formative evaluations for the three clinical simulation scenarios (AMI, BLS, and SDH) were performed through revised OSCEs, andSA factors were added to simulated scenarios to explore differences in SA levels under different scenarios. At the end of the semester, all students were evaluated on their summative performance in medical and surgical nursing, medical and surgical nursing labs, and medical and surgical nursing internships as well as OSCE performance under different simulated scenarios and situation awareness by the instructor of each class. Data were collected at the end of the semester through the student grade performance system.

### Research tools

#### Medical and surgical nursing courses

Medical and surgical nursing is a core competency course as well as a major subject in Taiwan’s national nursing examination. Students are taught to apply basic biomedical and scientific knowledge; assess and analyze patients’ physical, mental, spiritual, and sociocultural responses; provide suitable nursing measures; and accurately implement and apply relevant techniques to clinical practice. The experimental group received innovative instruction, and the control group received conventional instruction, as defined in Table 1.

**Table 1:** Experimental Group vs Control Group Syllabi

Type	Experimental group: Innovative instruction	Control group: Conventional instruction
Time	Every course unit follows a topic scenario, each session was 30–90 min long with a total instruction time of 188 h	
Instructor’s role	Teaching materials were made with innovative strategies and uploaded to a digital platform; intelligent tools and mobile applications were used to interact with students during class sessions	Only slide presentations and videos were used during class; interaction with students were limited to lecture and questions; no on-line materials were provided for students
Student’s role	Students could learn and interact with instructors through either on-line platform or mobile applications both during and after class	Students could only attend lectures during class sessions.
Teaching strategy	1. Lectures 2. Flipped classrooms 3. Group discussions 4. ITII: SPOCs, Zuvio, advanced simulator, smart Little Anne QCPR manikin, AED, Moodle, LINE 5. Practical: clinical simulation scenario 6. OSCE	1. Lectures 2. Group discussions 3. Multimedia: Slide presentations and videos
Lessons	1. Body fluid and electrolyte imbalance care 2. Respiratory system disease care and respiratory tract treatment 3. Cardiovascular disease care 4. Introduction to basic life-saving skills/advanced heart-saving surgeries 5. Shock, sepsis, and multiple organ failure care 6. Endocrine disorder care 7. Nervous system disease care 8. Urinary system disease care	1. Body fluid and electrolyte imbalance care 2. Cardiovascular disease care 3. Myocardial infarction and emergency care 4. First-aid cardiopulmonary resuscitation 5. Shock care 6. Endocrine system disease care 7. Nervous system disease care 8. Urinary system disease care
Assessment	Summative performance in medical and surgical nursing, medical and surgical nursing labs, and medical and surgical nursing internships as well as OSCE performance under different simulated scenarios and situational awareness	

#### Course development and design of clinical simulation scenario lesson plans

Course integration and development of clinical scenario lesson plans were accomplished in two stages, as per Thomas et al. [23] Clinical simulation scenario learning was then added, along with explorations based on the outcomes of ITII.

### Stage 1: integrating the medical–surgical and critical nursing courses

The medical and surgical nursing course development team was comprised of eight nursing instructors, three clinical nursing experts, and one clinical physician. They integrated the course syllabus, purpose, teaching strategies, teaching progress and content, performance evaluations, textbooks, and reference books of teaching courses originally aimed at fourth-year students of a 5-year continuous nursing program. The course development team also incorporated scenario simulations with ITII as a teaching strategy and established a critical nursing course using flipped classroom concepts and a revised OSCE.

### Stage 2: developing scenario simulation teaching and clinical scenario teaching templates with the ADDIE model

#### 1. Developing scenario simulation teaching and clinical scenario teaching templates

Appropriate clinical teaching templates were developed referencing the Human Patient Simulation Scenario Development Patient Case Template (HPSSDPCT) [24, 25] and the Template of Events for Applied and Critical Healthcare Simulation (TEACH Sim) [24] and using the Analysis, Design, Development, Implementation, and Evaluation (ADDIE) [26–28] teaching design model, while also introducing scenario simulation teaching into the lesson plans. First, in the Analysis phase, researchers analyzed the students, the curriculum, training tools and the learning environment. Then, based on results of the analyses, the Design phase employed SMART (Specific, Measurable, Achievable, Relevant, Timely) principles to formulate course syllabus, content set-up and objectives. Then in the Development phase, experts in clinical nursing and medicine would discuss over course content such as presentation, activities, interface design and course feedback, generating an instructor’s and a student’s manual. During the Implementation phase, plans from the previous phase were executed with programming design, script writing for simulation scenarios, and visual design. Lastly is Evaluation, to assess the effectiveness of course content and interface from the previous phases. Three KSA domains—knowledge (cognitive), skills (psychomotor), and attitude (affective)—were used to set learning priorities, key events, and target responses.

#### 2. Designing the clinical scenario simulation lesson plans

The medical and surgical nursing course development team formulated clinical scenario lesson plans based on five topics: percutaneous transluminal coronary angioplasty (PTCA) and stent placement care for patients with acute myocardial infarction (AMI); basic life support (BLS) and automated external defibrillator (AED) operation; subdural hemorrhage (SDH) care; applications of the advanced Apollo Simulator for patients with septic shock; and acute respiratory distress syndrome (ARDS) care.

Clinical nursing and medical experts in each medical field were invited to join the development team to discuss and revise the teaching scenarios. Next, two pilot tests were conducted, and the scenarios were revised again based on the test results to form the final version. Each class session was 30 to 90 min in length, depending on the topic scenario.

### OSCE checklist development and reliability and validity tests

OSCE scores are a measurement tool that deconstruct observable tasks to evaluate student performance, and so all OSCE checklists must be reliable and valid. Therefore, after the first drafts of the OSCE checklists for the three scenario simulation lesson plans in this study were completed, they were tested for face validity, content validity, and criterion validity [2].

#### 1. Creating the first draft of the checklists

OSCE checklists were created for PTCA care for patients with AMI, BLS and AED operation (henceforth “BLS), and SDH care. Both septic shock care and ARDS care were taught as part of the coursework but excluded from the OSCEs because they required the use of advanced simulators not available to the study team. The checklists itemized the required actions in the care procedure, and each checklist item was graded based on whether it was fully completed (2 points), partially completed (1 point), or not completed (0 points).

#### 2. Performing expert content validity tests

The first drafts of the three clinical scenario checklists were reviewed by 17 clinical experts and three fifth-year nursing students for appropriateness, clarity, conciseness, and wording, as well as content validity. Using the content validity index (CVI), each item of the evaluation survey was assigned 1 to 4 points on a 4-point Likert scale, wherein 4 was “very appropriate,” 3 was “appropriate,” 2 was “inappropriate,” and 1 was “very inappropriate.” Each checklist could receive up to a 100 total points. Each checklist item was assessed by a subject-matter expert, and for every checklist, the percentage of items that were scored “appropriate” or “very appropriate items” was calculated. Checklists were deemed to have a favorable validity index if their score was at least 80% [29].

The checklist evaluations and expert opinions were then organized into a summary table. Items that received 3 or 4 points were retained; items that received only 1 or 2 points or were considered unclear or badly-worded were either revised or deleted after the course development team considered the expert commentary.

#### 3. Reliability testing

The revised checklists developed for the three clinical scenario topics (AMI, BLS and AED, and SDH) were rated for inter-rater reliability using the Kendall coefficient of concordance. Six students were sampled for each topic, and the OSCEs were held in the clinical skills center and recorded on video. Four nursing instructors evaluated the performances according to the three revised checklists. The reliability of the three OSCE checklists were also evaluated using Cronbach’s α.

### Determination of SA levels

Research has shown that SA influences decision-making and is affected by factors such as knowledge, perception, task goals, degree of understanding, message deciphering, level of attention, and memory [9]. SA items were added to this study because the unpredictable nature of the care environment and complexity of patient care demand high levels of situational awareness. Two clinical design experts and three human-factors engineering experts were invited to the course development team meeting to discuss and determine the necessary SA of each item; the determination of SA levels was reached through consensus, and they were integrated into the OSCE checklists. The levels were determined as follows:

1. SA1 (perception): review, confirmation, basic interpretation, the execution of basic skills, and patient identification.
2. SA2 (comprehension): the interpretation of information, further treatments following observation, decisions and treatments based on SA1, and reports.
3. SA3 (projection): advanced interpretations, continued treatment, proposals of precaution and health education, decisions and treatments based on SA2, and reports.

### Application of ITII

Seven information technology tools were used in accordance with the course progress and clinical scenario topics:

#### 1. EWANT (SPOCs)

Based on the medical and surgical nursing course outlines, videos were recorded in school MOOC classrooms, edited and then uploaded to the Ministry of Education’s EWANT online learning platform to create SPOCs. The instructor released 20 learning topics based on course progress, and after receiving their account and password, the experimental group students could log in to the platform and engage in self-learning before class. Statistical information regarding student engagement and duration was collected from EWANT.

#### 2. Zuvio (interactive classrooms)

Zuvio is an e-learning platform that allows lesson preparation and extensive student-teacher interaction during class. The campus version of Zuvio was used in the clinical simulation courses. During class, the instructor presented course materials based on the scenario progress, which included videos, physician orders, electrocardiograms, and inspection reports. Students could watch the materials on their smartphones and engage in interactive activities in real-time, such as interpreting clinical data and answering questions. Zuvio was also used for post-class tests, reviews, and roll call.

#### 3. MOODLE (teaching platform)

The Moodle teaching platform (version 3.2.3+, build: 20170512) was used for its administrative and management functions, such as instructor announcements, tracking teaching progress, file storage, and teaching evaluations.

#### 4. LINE messaging app

All participants in the experimental group interacted with the instructors using the messaging app LINE, through which they also received messages, announcements, and reminders and participated in after-class discussions. Furthermore, all the clinical simulation scenario files were uploaded to the LINE group for students to download.

#### 5. High-fidelity wireless simulator

The high-fidelity wireless simulator used in this study was the Apollo Patient Simulator (CAE Healthcare, Canada), which has built-in batteries and gas compressors. During scenario lessons, the simulator’s “monitored” physiological data was projected onto the classroom screen while the Apollo Simulator presented the corresponding clinical symptoms, allowing students to perform physical examinations, interpret the preset physiological data to uncover patient problems, and implement appropriate nursing measures. The scenario topics that used this setup included professional nursing skills and knowledge for monitoring and assessing vital signs; measurement of central venous pressure and catheter care; endotracheal tube care; sputum suctioning; oxygen therapy use and efficacy assessment; urinary catheter care; assessment of states of consciousness; nasogastric tube care; and feeding methods.

#### 6. Little Anne QCPR

The “Little Anne QCPR” (Model number 123–01050, Laerdal) is an adult upper torso manikin that combines the detection and feedback on of high-quality CPR items such chest compression depth and speed, respiratory volume accuracy, etc. This smart teaching aid was used for teaching BLS skills, in the AED simulation scenarios, and in the revised OSCEs for testing student performance. When in use, the manikin was paired with a phone or tablet app to display and record operational results.

#### 7. AED trainer 3

The AED Trainer 3 by Philips was used in BLS and AED simulation scenario teaching and the student OSCEs. This model is compliant with the American Heart Association first-aid guidelines. The trainer’s first-aid procedures were configured by the instructors, and the trainer was connected to a simulator.

### Statistical analysis

The data were organized and analyzed using SPSS 23.0 (Armonk, NY: IBM Corp.). The statistical methods used included descriptive statistics, independent samples *t* tests (*p* < 0.05 was considered significant), Kendall coefficient of concordance, Cronbach’s α, and Pearson correlation coefficient analysis. Student performance was analyzed with descriptive statistics, including medical and surgical nursing, medical and surgical nursing labs, and medical and surgical nursing internships; independent samples *t* tests were used to compare differences in student performance between the experimental and control groups; Kendall coefficient of concordance was used to test inter-rater reliability; Cronbach’s α for reliability of the OSCE checklist; and Pearson correlation coefficient analysis was used to explore the correlation of the different groups to OSCE.

## Results

A total of 120 participants were recruited into the study: 61 participants into the experimental group and 59 into the control group. In the control group, one student withdrew from school during the clinical internship, so there were only 58 sets of clinical internship grades (missing value = 1).

### Reliability and validity analysis of the three OSCE checklists

The AMI OSCE checklist had a CVI of 0.981, and so four items were revised, one item was deleted, and 26 items were retained. The BLS and AED OSCE checklist had a CVI of 0.987, and so three items were revised and 25 items were retained. The SDH OSCE checklist had a CVI of 0.981, and so one item was revised and 13 items were retained.

The Kendall’s coefficient of concordance values of the three clinical scenarios are presented in Table 2 and indicated significant correlations among the nursing instructors’ evaluations, and therefore the inter-rater reliability and consistency of the checklists were deemed acceptable.

**Table 2:** Kendall Coefficient of Concordance

Topic	PTCA care for AMI patients	BLS and AED	SDH care
Number	4	4	4
Kendall’s W^a^ test	0.937	0.896	0.965
Chi square	18.731	17.923	19.307
Degree of freedom	5	5	5
Asymptotic significance*	0.002^*^	0.003^*^	0.002^*^

a. Kendall coefficient of concordance.

*p < 0.05

The Cronbach’s α values were 0.608 for the AMI checklist, 0.797 for the BLS checklist, and 0.761 for the SDH checklist, with the latter two achieving high reliability.

### Learning outcomes of the clinical simulation scenarios and ITII

#### Medical and surgical nursing course grades before and after intervention

Since the study started in fourth year, third-year course grades could serve as the baseline for both groups before the start of the courses, whereas fourth-year grades as one of the outcomes of the intervention. Therefore, comparison between the two groups were made at baseline (Year 3) and after intervention (Year 4), with the independent samples t test results shown in Table 3. While the two groups were not significantly different at baseline (Year 3) [t(61.59) = 1.229, *p* = 0.222, Cohen’s *d* = 0.22], there was a significant difference between the two groups after the intervention (Year 4) [t(61.59) = 2.392, *p* = 0.018, Cohen’s *d* = 0.46].

**Table 3:** Independent Samples t Tests of Participants’ Third- and Fourth-Year Medical and Surgical Nursing Grades

Variables	Experimental group (*n* = 61)		Control group (*n* = 59)		*t* value	df	*p*-value (two-tailed)
	M	SD	M	SD			
Total grade, Year 3	76.59	6.927	74.66	10.035	1.229	118	0.222
Total grade, Year 4	79.39	6.004	76.10	8.849	2.392	118	0.018

df, degree of freedom.

#### Correlation between clinical simulation scenarios and internship OSCE results

Correlations between the medical and surgical clinical internship scores and OSCE results were determined through Pearson correlation coefficients. Only the experimental group showed any significant difference between their OSCE score and their medical and surgical clinical internship score for BLS [r(61) = 0.301, *p* = 0.018], which showed a low, positive correlation (Table 4).

**Table 4:** Medical and Surgical Clinical Internship OSCE Scores: Pearson Correlation Coefficients Between Innovative and Conventional Teaching Methods

Variables	Experimental group (n = 61)		Control group (*n* = 59)	
	Pearson correlation coefficient	*p*-value (two-tailed)	Pearson correlation coefficient	*p*-value (two-tailed)
OSCE total	0.222	0.085	−0.059	0.657
OSCE_AMI	0.166	0.200	−0.151	0.259
OSCE_BLS	0.301	0.018	0.144	0.280
OSCE_SDH	0.073	0.577	−0.123	0.357

* *p* < 0.05

#### Clinical simulation scenarios and ITII on medical and surgical nursing lab scores and internship scores

The medical and surgical nursing lab scores and clinical internship scores of the experimental and control groups were compared using independent samples *t* tests. For the lab scores, the results indicated that the experimental group’s average scores were significantly higher than the control group’s average by 3.46 points [t(61.58) = 1.944, *p* = 0.048, Cohen^’^s *d =* 0.36] (Table 5). For the clinical internship scores, the results showed no significant differences between the two groups, with the experimental group outperforming the control group by only 0.04 points (Table 5).

**Table 5:** Medical and Surgical Nursing Lab Scores and Clinical Internship Scores for Innovative and Conventional Teaching Methods

Variables	Experimental group (n = 61)		Control group (*n* = 59/58)**		*t* value	df	*p*-value (two-tailed)
	M	SD	M	SD			
Lab scores	76.38	7.982	72.92	10.860	1.944	118	0.048
Clinical internship scores	80.02	3.253	79.98	4.224	−0.049	117	0.961

df, degree of freedom.

* *p* < 0.05

**The control group had 59 sets of lab scores and 58 sets of clinical internship scores

#### Influence of clinical simulation scenarios and ITII on OSCE formative evaluations

The total OSCE scores and AMI, BLS, and SDH scores of the experimental and control groups were compared using independent samples *t* tests. The results showed that the experimental group significantly outperformed the control in OSCE total scores [t(61.59) = 7.885, *p < 0.01,* Cohen^’^s *d = 1.44*], AMI scores [t(61.59) = 6.840, *p < 0.01,*Cohen^’^s *d = 1.25*], and SDH scores [t(61.59) = 6.469, *p < 0.01,* Cohen^’^s *d = 1.18*], the last by nearly 15 points (Table 6).

**Table 6:** Influence of Innovative and Conventional Teaching Methods on OSCE and the Three Topics

Variables	Experimental group (n = 61)		Control group (*n* = 59)		*t* value	df	*p*-value (two-tailed)
	M	SD	M	SD			
OSCE total	230.18	26.890	194.97	21.656	7.885	118	0.000**
AMI	84.49	15.802	67.42	11.030	6.840	118	0.000**
BLS	85.21	8.456	82.14	13.965	1.466	118	0.145
SDH	60.57	11.566	45.61	13.714	6.469	118	0.000**

df, degree of freedom.

* *p* < 0.05

** *p* < 0.001

#### Clinical simulation scenarios and ITII on SA among the three OSCE topics

The SA1, SA2, and SA3 scores for the three OSCE topics were compared using independent samples *t* tests. The results showed significant differences in AMI SA2 [t(61.59) = 5.171, *p < 0.01,* Cohen^’^s *d = 0.95*] and SA3 [t(61.59) = 8.989, *p < 0.01,* Cohen^’^s *d =* 1.64]; in BLS SA1 [t(61.59) = − 2.215, *p* = 0.029, Cohen^’^s *d* = 0.40], SA2 [t(61.59) = − 2.146, *p* = 0.034, Cohen^’^s *d* = 0.39], and SA3 [t(61.59) = 8.982, *p < 0.01,* Cohen^’^s *d* = 1.63]; and in SDH SA1 [t(61.59) = 4.395, *p < 0.01,* Cohen^’^s *d* = 0.80] and SA2 [t(61.59) = 6.296, *p < 0.01,* Cohen^’^s *d* = 1.14]. Except for BLS SA1 and SA2, in which the control group scored higher, the experimental group outperformed the control group across the board, especially in BLS SA3 (Table 7).

**Table 7:** Innovative and Conventional Teaching Methods on OSCE SA

Variables	Experimental group (n = 61)		Control group (*n* = 59)		*t* value	df	*p*-value (two-tailed)
	M	SD	M	SD			
AMI_SA1	23.92	4.394	24.93	2.599	−1.532	118	0.128
AMI_SA2	30.98	6.896	25.00	5.702	5.171	118	0.000**
AMI_SA3	29.46	7.435	17.15	7.561	8.989	118	0.000**
BLS_SA1	44.52	4.660	47.19	8.104	−2.215	118	0.029*
BLS_SA2	22.72	3.984	24.47	4.932	−2.146	118	0.034*
BLS_SA3	17.97	2.840	10.47	5.841	8.982	118	0.000**
SDH_SA1	21.66	3.803	17.95	5.332	4.395	118	0.000**
SDH_SA2	25.89	5.404	16.07	10.869	6.296	118	0.000**
SDH_SA3	13.00	6.807	11.63	6.526	1.127	118	0.262

df, degree of freedom.

* *p* < 0.05

** *p* < 0.001

## Discussion

### Building clinical scenario lesson plans for medical and surgical nursing

In this study, course integration and scenario development were created by a medical and surgical nursing course development team, through nursing classes, as well as including a cross-professional team of clinical nurses and physicians. Combining the ADDIE model [26] with the HPSSDPCT and Benishek et al.’s TEACH Sim template [24] to construct the simulation scenario lesson plans was highly beneficial. In the construction process, reaching a team consensus on the guidelines, technical handbooks, textbooks, and clinical practices was challenging; industry–academia differences had to be reduced or addressed during this process. The scenarios had to be designed to resemble clinical practices as much as possible, and purposeful simulation designs were expected to effectively improve the structure, process, or results of the course goals and/or the institution. Furthermore, the scenario materials were based on actual case files from St. Martin De Porres Hospital and the Internet and were confirmed by the course development team and clinical specialist physicians. In accordance with Lioce et al.’s proposition, the simulation-based experiences were specifically designed to achieve confirmed goals [30]. The standardized simulation designs provided a framework for building effective simulation-based experiences and favorable evidence for adult learning fields, education, teaching design, clinical care standards, evaluations, and simulations.

### Developing OSCE instruments and their reliability and validity

The OSCEs were developed through integrated scenario building and were based on McWilliam and Botwinski’s guidelines [31]: 1) case scenarios must be built by experts and instructors within the topic to test the effectiveness of the competency being evaluated; and 2) if more than one professional field is being tested, then experts in all of the relevant disciplines should work together while keeping current conditions in mind and adhering to clinical procedures. The development of the OSCE checklists was based on teaching goals and the research purpose but did not follow the Angoff scoring method. Instead, the percentage of each checklist item was converted into points, and in accordance with Harden et al. and Wessel et al., the checklists were then expanded to include more fields for addressing the shortcomings of binomial options [32, 33]. Furthermore, based on Fox et al.’s three-item scoring method, scores were assigned for fully completed (2 points), partially completed (1), and not completed at all (0) [34], with the tasks and number of actions to be completed were clearly defined. Moreover, after a consensus had been reached on the scenario outlines of each topic, the content validity was tested by consulting clinical specialist physicians, specialist nurses, hospital senior nurses, infection control nurses, supervisors, and four nursing students. Rushforth argued that evaluation standards must be developed for any OSCE [2]; in this study, face validity, content validity, and criterion validity were all tools for scoring construction, and the three OSCE checklists scored CVI of 0.981 to 0.987. Using Kendall’s W, the revised checklists showed significant inter-rater reliability among three sets of six unrepeated students and four examiners, this indicating high levels of both reliability and consistency. Furthermore, the Cronbach’s α values of the AMI, BLS, and SDH checklists’ reliability were between 0.608 and 0.797, with the BLS and SDH checklists reaching high reliability. The Cronbach’s α values in this study were all comparable to or slightly higher than other values found in the literature [34–38], and the internal rate of return was probably higher because multiple experts were involved in all stages; the lesson plan topics were focused; and the checklist items were objective, highly detailed, and rigorous. It should also be noted that the examiners were all also members of the course development team. Previous studies have had topics with broader scopes, which may have affected the process of designing the OSCE checklist, applied content, operations, and number of testing stations, producing the relatively lower levels of reliability and validity.

### Implementing innovative teaching practices: clinical simulation scenarios and ITII

The scenario building process in this study referred to the work of Bambini, Lioce et al., and Harrington and Simon [25, 30, 39]. The scenarios were based on the writers’ own experience of patient conditions and were supplemented and fleshed out using accumulated academic knowledge to improve their clinical integrity. Bambini suggested adjusting the complexity of the simulation based on the learner’s level and that complicated scenarios should be separately built for more experienced and senior workers [39]. Scenario lesson plans for nursing students should consider the students’ academic abilities and the corresponding available data to approximate clinical practices and adjust complexity as needed. As a result, the developed OSCE checklists can achieve high reliability and validity.

A combination of high-fidelity simulation equipment, actors (a.k.a. standardized participants or “SPs”), were used in the simulation scenario lessons, as per Willhaus [40]. The venues used in the teaching process were equipped with standard ward equipment, patient units, simulated patients, basic medical facilities, tools, nursing carts, and emergency carts. The simulation lesson plans were ranged from simple to extremely complex, and the applications of the Apollo Simulator ranged from BLS to septic shock patient care. The simulation equipment varied greatly in function and was chosen based on the teaching goals and the desired outcomes of the scenario simulations, to ensure optimal operation and the adherence to key design criteria. As Childs and Sepples noted, as the construction and implementation of simulation labs require more time than conventional teaching methods do, under budget constraints, the number of advanced simulators – which are more expensive and complex – could be insufficient [41]. Therefore, the use of OSCEs was dropped from septic shock patient care in this study.

All the scenario materials were pre-built into the Zuvio interactive university classes, and students used their smartphones to watch the materials and answer questions according to the scenario. Because the scenario lessons consume a considerable amount of class time, SOPCs were introduced with flipped classroom formats and integrated into blended learning. Before the scenario lessons, students used their extracurricular time to prepare, which helped with the insufficient class times.

ITII covers the instructors’ teaching activities, students’ learning activities, teaching preparation, and classroom management [42]. Although studies have verified the educational advantages of incorporating technology solutions like ITII, nursing education still largely uses conventional methods. This study suggests that the instructors’ own technological literacy is one of the keys to promoting the use of technology in nursing education, which is consistent with the findings of Yeh et al. and Xu and Chen [43, 44]. How educational institutions cultivate instructors to possess relevant knowledge and competencies and maintain pace with technological development to adapt to new forms of education remains a critical issue.

SPOCs, rather than MOOCs, were used in this study, because the implementation of the latter carries some challenges (such as a high dropout rate) and also because the literature has already reported the advantages of replacing MOOCs with SPOCs [45–51]. Prior to beginning the course, students were required take the EWANT online preparatory course, which is a flipped classroom and adds practical courses for the simulated scenario lessons. The findings of this study indicate that SPOCs support the effectiveness of small-scale blended learning, allowing students to have more comprehensive and in-depth learning experiences while simultaneously providing instructors with flexible and feasible teaching models. Such models could help instructors understand students’ learning needs and behaviors using learning hours, achievement rates, and formative and summative evaluations.

### The effectiveness of introducing innovative teaching

#### Influence of innovative teaching on OSCEs

The experimental group outperformed the control group in terms of average and individual-subject OSCE scores across the board, particularly in SDH, by nearly 15 points, and with significant differences between their total OSCE scores and their AMI and SDH subject scores. This suggests that interventional clinical simulation scenarios and ITII had favorable effects on OSCE formative evaluation scores. No significant difference was found between the two groups for BLS, possibly because this topic involves more basic skills than complex ones. Hu et al. [52] compared between flipped learning and conventional instruction for hyperthyroidism knowledge and care skills among medical interns and found no difference in the knowledge component, while there was a significant difference in clinical case analysis where students in flipped learning performed better than those in conventional instruction. Comparing third-year summative evaluation to fourth-year first-semester scores in medical and surgical nursing, moderate and low levels of correlation were exhibited only among the experiment group’s medical and surgical nursing lab and clinical internship scores and total scores on the revised OSCEs, whereas the control group showed no significant differences at all. This implies that the innovative instruction did make a difference on the total scores on the revised OSCEs, and also that introducing innovative teaching methods could lead to nursing students performing better on OSCE formative evaluations than would conventional ones. The revised OSCE was used in this study as the formative evaluation for subjects, not only to audit the students’ performance but also to improve them. This improvement was possibly achieved through providing feedback and adjusting in-progress teaching and learning, as well as by developing intervention measures, thus achieving effective learning [53–55].

#### Whether SA is affected by innovative and conventional teaching

Among the three topics, the experimental and control groups demonstrated significant differences in SA2 and SA3 in AMI; in SA1, SA2, and SA3 in BLS; and in SA1 and SA2 in SDH. Where the differences were significant, the experimental group scored higher than the control group, except for SA1 and SA2 in BLS, where the control group scored higher. This result implies that innovative instruction could help students develop significantly different levels of SA. Typically speaking, new nurses should have high levels of SA1 and SA2 in most clinical scenarios, as they are novice professionals and advanced learners, and will only develop a certain degree of SA3 after achieving professional competence.

However, the experimental group did not develop consistent SA differences in all three topics, which may be because of the different difficulty levels of each topic. Curl et al. [56] found different effects by integrated simulation on different topics in nursing education. In BLS, which is comparatively simpler than AMI and SDH, the two groups exhibited significant differences in all three SA levels, but the control group demonstrated slightly higher levels of SA1 and SA2 than the experimental group. Whether this was caused by a possible greater familiarity with BLS requires further discussion. As for the other two topics, AMI is moderately difficult, with the two groups demonstrating significant differences in SA2 and SA3, implying that SA1 was basic knowledge for nursing students; and SDH is an even more difficult topic, with significant differences between the two groups’ SA1 and SA2 only.

#### Whether innovative teaching and conventional teaching methods affect academic learning effectiveness

No significant differences were found in the third-year total scores between the experimental and control group, implying that both groups were at around the same level in their medical and surgical nursing summative evaluations, possibly due to all receiving the same conventional instruction. However, significant differences were observed in the total scores of the first semester of their fourth year, with the experimental group scoring higher by an average of 3.29 points. This suggests that the innovative instruction produced significant improvements in their academic grades. In lab grades, the experimental group significantly outperformed the control group by 3.46 points, implying that innovative instructions were superior to conventional ones for improving short-term lab performance during the one semester of intervention. Consistent with past studies [21, 22], innovative instruction was found to boost student motivation and performance in healthcare education. However, there were no significant differences between the groups’ clinical internship grades, which was attributed to the short time invested in the innovative teaching experiment (only one semester, a total of approx.. 188 in-class hours). Harrington et al. [57] compared student performance between flipped learning and conventional instruction by testing three times during the semester and found no difference among the groups, demonstrating that the differences were not easily detected within a short span of time, especially when the course content was mostly practical. Greater differences between methods may be produced if clinical simulation scenarios and ITII could be utilized for a longer period of time.

## Conclusion and limitations

### Conclusion

This study developed a simulated instruction system with clinical scenario template for nursing education based on the ADDIE model through interdisciplinary collaboration with nursing education, clinical nursing and medicine to make scenarios as close to real-life as possible, as well as OSCE checklists based on the aforementioned simulated clinical scenario through consensus after many discussions to ensure objectivity and attention to details. The AMI, BLS, SDH assessment had Cronbach’s α between 0.608–0.797 and CVI of 0.981 or above, demonstration the OSCE checklists to be reliable.

For student performance, there was no significant difference between the experimental and control groups before the course, demonstrating their similarities, but student performance for the knowledge component in the experimental group (innovative instruction) was better than the control group (conventional instruction) after the courses, demonstrating the effectiveness of innovative instruction to boost knowledge-based learning within a short amount of time. There was no significant difference in the practical component, possibly due to the more complex and varying nature of practical skills in which differences could not be detected within a short span of time.

In terms of OSCE performance, the experimental group performed better than the control group in overall OSCE, AMI and SDH, demonstrating the effectiveness of innovative instruction on boosting clinical care skills. However, since BLS content is more knowledge-based, there was no significant difference in OSCE between the two groups.

On the other hand, under different levels of situational awareness, student performance was related to task difficulty. Therefore in the highly-difficult SDH tasks, there was a difference between the experimental and control groups on the OSCE for SA1 and SA2, namely, the experimental group performed better in the perception and comprehension levels, but there was no significant difference in the projection level. In the medium-difficult AMI tasks, there was a significant difference between the groups in SA2 and SA3 of OSCE, showing that students in innovative instruction performed better in comprehension and projection for AMI tasks than those in conventional, but there was no difference in perception between the groups. For the easier BLS tasks, the experimental group performed better in SA3 but worse in SA1 and SA2 compared to the control group. This might suggest that conventional instruction was helpful with perception and comprehension in BLS tasks, but the projection level could be improved with innovative instruction.

Overall, innovative instruction could help boost performance in knowledge-based content and OSCE performance, and the different teaching methods affect different levels of situational awareness in practical tasks. This study could serve as a reference for future nursing education research, as well as recommendation for instructors of clinical nursing or medicine.

### Limitations and future research

The participants in this study came from purposive sampling in a five-year junior college program, which was not the main nursing degree program in Taiwan. Future participants could be recruited from, and comparisons made with other types of nursing degree programs such as two-year technical programs and bachelor’s degree programs for broader representation and better objectivity. Clinical expertise in this study were all provided from medical specialists, clinical supervisors, head nurses, and nurse practitioners at a single institution (St. Martin De Porres Hospital). Future collaborations with other institutions from different levels of care could be explored to expand and optimize the design for OSCE checklists. The innovative teaching methods were conducted in a hybrid manner, making explorations of any single interventions unfeasible. Future studies could evaluate the effects of single elements in teaching methods.

Due to constraints in resources and time, the innovative teaching methods were applied for one single semester and only modest modifications were made to existing OSCE checklists. Extensive re-development of the entire medical-surgical nursing curriculum was recommended. With the proliferation of information technology-based applications in teaching methods for nursing education, more proficiency in more recent technologies, as well as inter-disciplinary communication are needed in the continuing education of instructors to optimize nursing education.

## Data Availability

The data presented in this study are available on request from the corresponding author. The data are not publicly available due to legal restrictions imposed by the government of Taiwan in relation to the “Personal Information Protection Act”.
